# GLP-1 RA Use and Survival Among Older Adults With Cancer and Type 2 Diabetes

**DOI:** 10.1001/jamanetworkopen.2025.21887

**Published:** 2025-07-18

**Authors:** Rotana M. Radwan, Ying Lu, Hao Dai, Thomas J. George, Yi Guo, Jingchuan Guo, Jiang Bian

**Affiliations:** 1 Department of Pharmaceutical Outcomes and Policy, College of Pharmacy, University of Florida, Gainesville; 2 Department of Health Outcomes and Biomedical Informatics, College of Medicine, University of Florida, Gainesville; 3 Department of Medicine, Division of Hematology and Oncology, College of Medicine, University of Florida, Gainesville; 4 Regenstrief Institute, Indianapolis, Indiana; 5 Department of Biostatistics & Health Data Science, Indiana University School of Medicine, Indianapolis; 6 Melvin and Bren Simon Comprehensive Cancer Center, Indiana University, Indianapolis

## Abstract

This cohort study evaluates whether older adults with type 2 diabetes and cancer have improved survival when taking glucagon-like peptide-1 receptor agonists (GLP-1RAs) compared with sodium-glucose cotransporter-2 inhibitors and dipeptidyl peptidase-4 inhibitors.

## Introduction

The potential role of glucagon-like peptide-1 receptor agonists (GLP-1RAs) in oncology has drawn interest due to their ability to lower glucose, adiposity, insulin resistance, and systemic inflammation—factors associated with cancer progression.^1^ The impact of glucose-lowering drugs (GLDs) on cancer survival remains inconclusive. Cohort studies of patients treated with metformin, insulin, or sulfonylureas reported mixed survival estimates that varied by drug and cancer type.^2^ Among newer GLDs, sodium-glucose cotransporter-2 inhibitors (SGLT2is) have been associated with improved survival compared with dipeptidyl peptidase-4 inhibitors (DPP4is).^3^ Although GLP-1RAs have been associated with a lower incidence of some cancers,^4^ their effect on survival remains unclear. This study evaluates whether GLP-1RA use is associated with improved survival in patients with cancer and type 2 diabetes (T2D).

## Methods

We conducted a retrospective cohort study using 2013 to 2020 Medicare data to examine the association between GLP-1RA use (compared with SGLT2i and DPP4i use) and all-cause mortality (eTable in Supplement 1). Eligible patients were aged 66 years and older, had T2D and 1 of 9 cancers (thyroid, pancreatic, bladder, colorectal, lung, kidney, breast, endometrial, or prostate), initiated 1 of the study drugs, and survived at least 1 year after cancer diagnosis. Patients were followed up from first drug prescription (index date) until death or December 31, 2020.

Baseline covariates included sociodemographic characteristics, comorbidities, and comedications. To reduce confounding, we conducted 1:1 propensity score matching. Cox proportional hazards models were used to estimate hazard ratios (HRs) and 95% CIs under an intention-to-treat framework. Subgroup analyses evaluated effect modification by age, sex, race and ethnicity, obesity, and cancer type. To assess potential unmeasured confounding, E-values were calculated. All analyses were conducted using SAS version 9.4 (SAS Institute), with a 2-sided *P* < .05 considered statistically significant. The study followed STROBE reporting guidelines and was approved by the University of Florida IRB with a waiver of informed consent. Analyses were conducted from January through February 2025.

## Results

The GLP-1RA vs SGLT2i cohort included 2553 matched pairs (mean [SD] age 73.9 [5.5] years; 56.0% male) with a median (IQR) follow-up of 1.65 (0.76-2.76) years. The GLP-1RA vs DPP4i cohort included 2564 matched pairs (mean [SD] age, 74.1 [5.6] years; 53.1% male), with a median (IQR) follow-up of 1.73 (0.83-2.79) years (Table).

**Table.** Baseline Characteristics of GLP-1RA vs SGLT2i Cohort and GLP-1RA vs DPP4i Cohort After 1:1 Propensity Score Matching^a^

Characteristic	GLP-1RA vs SGLT2i Cohort			GLP-1RA vs DPP4i Cohort		
	No. (%)		SMD	No. (%)		SMD
	GLP-1RA (n = 2553)	SGLT2i (n = 2553)		GLP-1RA (n = 2564)	DPP4i (n = 2564)	
Age, mean (SD), y	73.9 (5.6)	73.9 (5.5)	−0.0036	74.0 (5.7)	74.1 (5.6)	−0.0332
Race/ethnicity^b^						
Hispanic	182 (7.1)	190 (7.4)	0.0000	176 (6.9)	177 (6.9)	0.0000
Non-Hispanic Black	215 (8.4)	211 (8.3)		221 (8.6)	237 (9.2)	
Non-Hispanic White	2035 (79.7)	2040 (79.9)		2055 (80.1)	2058 (80.3)	
Other	121 (4.7)	112 (4.4)		112 (4.4)	92 (3.6)	
Sex						
Female	1148 (45.0)	1101 (43.1)	0.0101	1210 (47.2)	1196 (46.6)	−0.0062
Male	1405 (55.0)	1452 (56.9)		1354 (52.8)	1368 (53.4)	
Year of enrollment						
2014	57 (2.2)	62 (2.4)	0.0305	61 (2.4)	67 (2.6)	0.0309
2015	64 (2.5)	64 (2.5)		62 (2.4)	69 (2.7)	
2016	61 (2.4)	60 (2.4)		71 (2.8)	70 (2.7)	
2017	562 (22.0)	566 (22.2)		603 (23.5)	611 (23.8)	
2018	554 (21.7)	557 (21.8)		625 (24.4)	643 (25.1)	
2019	625 (24.5)	610 (23.9)		593 (23.1)	597 (23.3)	
2020	630 (24.7)	634 (24.8)		549 (21.4)	507 (19.8)	
Medicare and Medicaid dual eligibility	417 (16.3)	415 (16.3)	0.0002	448 (17.5)	476 (18.6)	−0.0098
Low-income subsidy	479 (18.8)	479 (18.8)	−0.0035	523 (20.4)	547 (21.3)	−0.0118
Clinician specialty						
Family practice or Internal medicine	1680 (65.8)	1683 (66.0)	0.0000	1699 (66.3)	1690 (65.9)	0.0365
Endocrinology	375 (14.7)	361 (14.1)		373 (14.5)	378 (14.7)	
Nurse practitioner or physician assistant	498 (19.5)	509 (19.9)		492 (19.2)	496 (19.3)	
Region						
West	267 (10.5)	268 (10.5)	0.0000	252 (9.8)	249 (9.7)	0.0000
Midwest	418 (16.4)	424 (16.6)		430 (16.8)	426 (16.6)	
Northeast	449 (17.6)	427 (16.7)		463 (18.1)	434 (16.9)	
Southwest	204 (8.0)	208 (8.1)		202 (7.9)	216 (8.4)	
Southeast	1215 (47.6)	1226 (48.0)		1217 (47.5)	1239 (48.3)	
Diabetes-related conditions						
Diabetes retinopathy	276 (10.8)	268 (10.5)	0.0031	274 (10.7)	258 (10.1)	0.0035
Diabetic neuropathy	766 (30.0)	766 (30.0)	0.0052	820 (32.0)	821 (32.0)	0.0003
Peripheral vascular disease	546 (21.4)	560 (21.9)	0.0001	570 (22.2)	604 (23.6)	−0.0038
Hypoglycemia	54 (2.1)	49 (1.9)	0.0047	68 (2.7)	61 (2.4)	−0.0080
Hyperglycemic emergency	16 (0.6)	18 (0.7)	0.0029	16 (0.6)	20 (0.8)	0.0009
GLD use at baseline						
No. of GLD use						
Insulin	696 (27.3)	679 (26.6)	0.0268	789 (30.8)	779 (30.4)	0.0248
No GLD (excluding insulin)	301 (11.8)	283 (11.1)		293 (11.4)	283 (11.0)	
1 GLD (excluding insulin)	894 (35.0)	919 (36.0)		846 (33.0)	862 (33.6)	
≥2 GLDs (excluding insulin)	662 (25.9)	672 (26.3)		636 (24.8)	640 (25.0)	
Metformin	1700 (66.6)	1720 (67.4)	−0.0104	1653 (64.5)	1659 (64.7)	0.0061
Sulfonylureas	1045 (40.9)	1061 (41.6)	−0.0078	1050 (41.0)	1029 (40.1)	−0.0132
DPP4i or SGLT2i	214 (8.4)	235 (9.2)	−0.0077	13 (0.5)	11 (0.4)	0.0050
Thiazolidinediones	211 (8.3)	203 (8.0)	−0.0076	184 (7.2)	189 (7.4)	−0.0052
Meglitinides	60 (2.4)	53 (2.1)	−0.0007	60 (2.3)	54 (2.1)	−0.0037
Comorbidities						
Acute myocardial infarction	159 (6.2)	166 (6.5)	0.0043	176 (6.9)	178 (6.9)	−0.0016
ADRD	240 (9.4)	241 (9.4)	0.0021	291 (11.3)	312 (12.2)	−0.0205
Atrial fibrillation	457 (17.9)	476 (18.6)	0.0034	490 (19.1)	510 (19.9)	−0.0028
Cataract	1609 (63.0)	1636 (64.1)	0.0010	1647 (64.2)	913 (35.6)	−0.0117
Chronic kidney disease	1848 (72.4)	1845 (72.3)	0.0122	1891 (73.8)	1651 (64.4)	0.0015
COPD	772 (30.2)	786 (30.8)	−0.0009	836 (32.6)	860 (33.5)	−0.0206
Chronic heart failure	811 (31.8)	830 (32.5)	−0.0032	875 (34.1)	911 (35.5)	−0.0183
Glaucoma	647 (25.3)	645 (25.3)	0.0103	657 (25.6)	679 (26.5)	−0.0040
Hip or pelvic fracture	32 (1.3)	32 (1.3)	−0.0004	42 (1.6)	50 (2.0)	−0.0061
Ischemic heart disease	1590 (62.3)	1600 (62.7)	0.0025	1613 (62.9)	1638 (63.9)	−0.0138
Osteoporosis	410 (16.1)	404 (15.8)	0.0001	437 (17.0)	443 (17.3)	−0.0201
Rheumatoid arthritis or osteoarthritis	1646 (64.5)	1639 (64.2)	0.0061	1710 (66.7)	1729 (67.4)	−0.0054
Stroke or TIA	348 (13.6)	341 (13.4)	−0.0051	377 (14.7)	379 (14.8)	−0.0099
Anemia	1632 (63.9)	1621 (63.5)	0.0054	1721 (67.1)	1747 (68.1)	−0.0047
Asthma	433 (17.0)	449 (17.6)	−0.0006	467 (18.2)	493 (19.2)	−0.0044
Hyperlipidemia	2449 (95.9)	2449 (95.9)	−0.0006	2463 (96.1)	2467 (96.2)	0.0059
Benign prostate hyperplasia	792 (31.0)	823 (32.2)	−0.0052	787 (30.7)	814 (31.7)	−0.0075
Hypertension	2453 (96.1)	2451 (96.0)	−0.0041	2469 (96.3)	2457 (95.8)	0.0002
Acquired hypothyroidism	850 (33.3)	830 (32.5)	0.0063	875 (34.1)	860 (33.5)	−0.0055
Inflammatory bowel disease	38 (1.5)	36 (1.4)	−0.0067	35 (1.4)	31 (1.2)	0.0000
Obesity	1005 (39.4)	987 (38.7)	0.0110	1057 (41.2)	1049 (40.9)	0.0152
Depression	989 (38.7)	973 (38.1)	0.0055	1071 (41.8)	1101 (42.9)	0.0016
Medications						
ACEI	887 (34.7)	905 (35.4)	−0.0038	910 (35.5)	904 (35.3)	0.0003
ARB	979 (38.3)	972 (38.1)	0.0045	957 (37.3)	944 (36.8)	0.0047
β Blockers	1333 (52.2)	1319 (51.7)	0.0022	1368 (53.4)	1371 (53.5)	−0.0114
Calcium channel blockers	932 (36.5)	919 (36.0)	0.0044	961 (37.5)	950 (37.1)	0.0026
Diuretics	1013 (39.7)	999 (39.1)	0.0059	1097 (42.8)	1078 (42.0)	−0.0093
Opioids	842 (33.0)	844 (33.1)	0.0044	920 (35.9)	933 (36.4)	0.0030
Antibiotics	456 (17.9)	428 (16.8)	0.0067	469 (18.3)	453 (17.7)	−0.0059
Statins	2006 (78.6)	2001 (78.4)	0.0034	2015 (78.6)	2001 (78.0)	0.0004
Antipsychotics	34 (1.3)	28 (1.1)	0.0094	35 (1.4)	40 (1.6)	−0.0036
NSAIDs	517 (20.3)	542 (21.2)	−0.0002	542 (21.1)	533 (20.8)	0.0026
Oral steroids	1095 (42.9)	1085 (42.5)	0.0015	1114 (34.4)	1124 (43.8)	0.0064
Antiplatelets	52 (2.0)	46 (1.8)	0.0030	53 (2.1)	63 (2.5)	−0.0058
Aldosterone receptor antagonists	177 (6.9)	174 (6.8)	0.0062	168 (6.6)	180 (7.0)	−0.0004
Anticoagulants	419 (16.4)	422 (16.5)	0.0052	439 (17.1)	465 (18.1)	−0.0052
Immunosuppressants	<11^c^	<11^c^	0.0028	<11^c^	<11^c^	−0.0054
TNF inhibitors	<11^c^	<11^c^	−0.0009	<11^c^	<11^c^	−0.0073
Antidepressants	882 (34.5)	865 (33.9)	0.0081	934 (36.4)	963 (37.6)	0.0020

Abbreviations: ACEI, angiotensin-converting enzyme inhibitor; ADRD, Alzheimer disease and related dementias; ARB, angiotensin receptor blockers; COPD, chronic obstructive pulmonary disease; DPP4i, dipeptidyl peptidase-4 inhibitor; GLD, glucose-lowering drug; GLP-1RA, glucagon-like peptide-1 receptor agonist; NSAID, nonsteroidal anti-inflammatory drug; SGLT2i, sodium-glucose cotransporter-2 inhibitors; SMD, standardized mean difference; TIA, transient ischemic attack; TNF, tumor necrosis factor.

^a^ All covariates were collected during 12 months before or on the index date.

^b^ Race and ethnicity were obtained from the Medicare Beneficiary Summary File, which uses data collected by the Social Security Administration and the Centers for Medicare & Medicaid Services, primarily through self-report at the time of enrollment. Original categories included: American Indian or Alaska Native, Asian or Pacific Islander, Black, Hispanic, White, and other or unknown. For this study, we recategorized these into 4 groups: Hispanic, non-Hispanic Black, non-Hispanic White, and other, which included beneficiaries identified as American Indian or Alaska Native, Asian or Pacific Islander, or other or unknown. Race and ethnicity were assessed to examine differences in clinical outcomes across populations.

^c^ Cell sizes less than 11 are suppressed in accordance with Centers for Medicare & Medicaid Services data use agreement requirements.

Mortality risk did not differ significantly between GLP-1RA and SGLT2i users (HR, 1.03; 95% CI, 0.85-1.23). In contrast, GLP-1RA use was associated with significantly lower mortality than DPP4i use (HR, 0.60; 95% CI, 0.51-0.70). Kaplan-Meier curves depicting cumulative mortality incidence are shown in the Figure. The E-value was 1.21 for GLP-1RA vs SGLT2i and 2.73 for GLP-1RA vs DPP4i.

**Figure.**
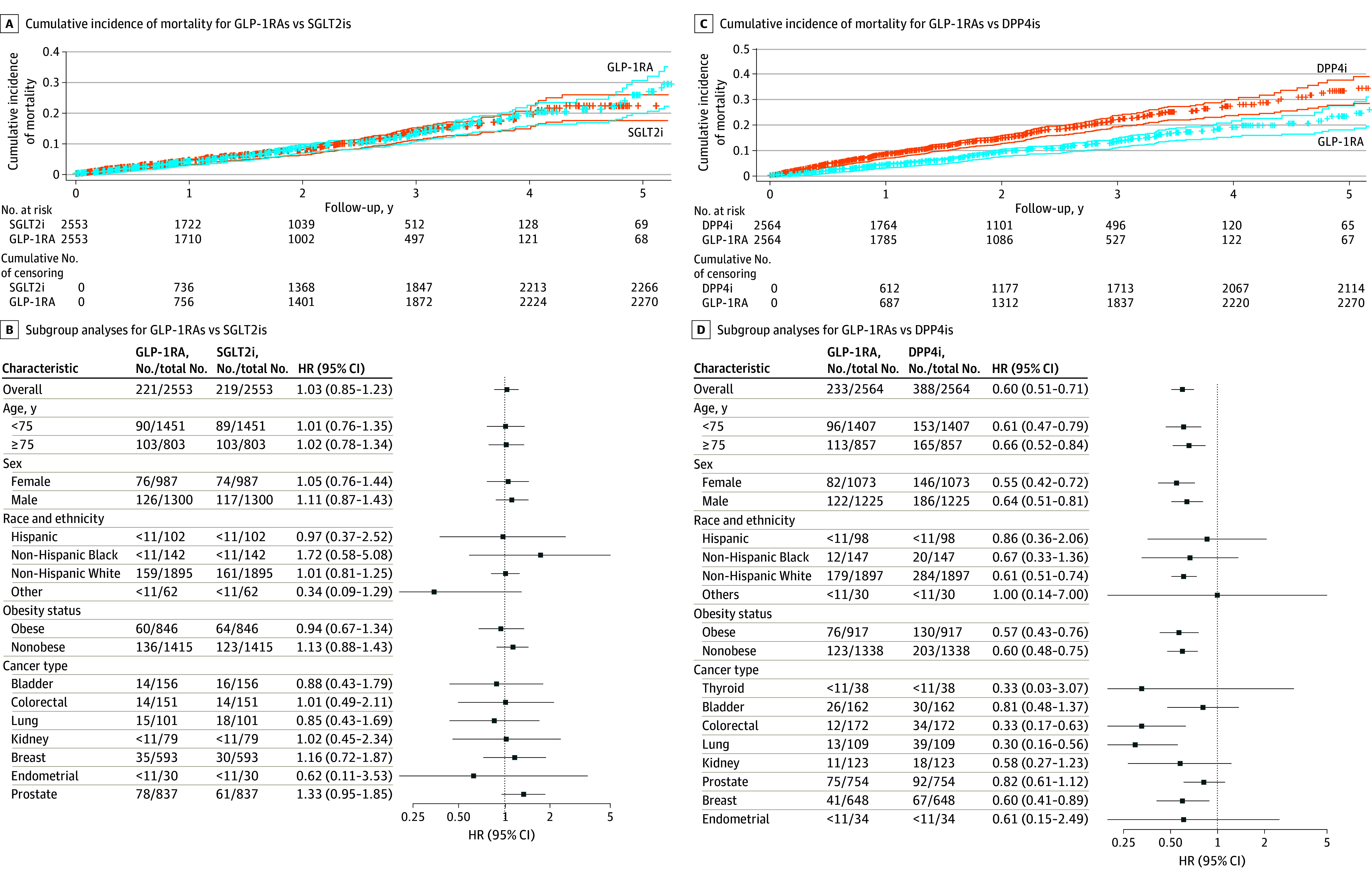
Cumulative Incidence of Mortality and Subgroup Analyses Among Patients With Cancer and Type 2 Diabetes Receiving Glucagon-Like Peptide-1 Receptor Agonists (GLP-1RAs) vs Sodium-Glucose Cotransporter-2 Inhibitors (SGLT2is) or Dipeptidyl Peptidase-4 Inhibitors (DPP4is) Lines represent cumulative incidence with 95% CI; crosses indicate censored patients. HR indicates hazard ratio.

Subgroup analyses revealed no significant differences between GLP-1RAs and SGLT2is. However, the survival benefit of GLP-1RAs over DPP4is remained consistent across age, sex, non-Hispanic White race, and several cancer types (colorectal, lung, and breast) (Figure).

## Discussion

In older patients with cancer and T2D, GLP-1RA use was associated with lower all-cause mortality compared with DPP4i use, with no significant difference relative to SGLT2i. The survival benefit over DPP4i persisted across age, sex, non-Hispanic White race, obesity status, and several cancer types (colorectal, lung, and breast).

A recent study^5^ found that high intertumoral GLP-1R expression predicts longer survival in some cancers (eg, bladder) but shorter in others (eg, cervical), highlighting the heterogeneity of GLP-1R signaling. While causality cannot be inferred, our study adds novel evidence on the comparative effectiveness of GLP-1RAs. The observed survival differences may reflect drug class–specific systemic effects. Although both GLP-1RAs and DPP4is target the incretin pathway, GLP-1RAs demonstrate greater reductions in hemoglobin A_1c_ levels and body weight, improved β-cell and cardiac function, and lower albuminuria.^6^ These effects may reduce cancer progression by mitigating hyperinsulinemia and inflammation, while also lowering overall mortality through broader health improvements. Study limitations include potential residual confounding and several underpowered subgroup analyses due to small sample sizes, limiting the ability to detect meaningful differences. Clinical trials are needed to confirm the role of GLP-1RAs in cancer survivorship.

## Notes

Supplement 1. **eTable.** Exposures of Interest and Comparison

Supplement 2.
Data Sharing Statement
