# Supplementary material for: GLP-1 RA Use and Survival Among Older Adults With Cancer and Type 2 Diabetes
Source: JAMA Netw Open. 2025 Jul 18;8(7):e2521887. doi: 10.1001/jamanetworkopen.2025.21887 (PMC12274974; doi:10.1001/jamanetworkopen.2025.21887)
Supplement: Supplement 1. — eTable. Exposures of Interest and Comparison [file jamanetwopen-e2521887-s001.pdf]

Supplemental Online Content

Radwan RM, Lu Y, Dai H, et al. GLP-1 RA Use and survival among older adults with cancer and type 2 diabetes. *JAMA Netw Open.* 2025;8(7):e2521887.
doi:10.1001/jamanetworkopen.2025.21887

eTable. Exposures of Interest and Comparison

This supplemental material has been provided by the authors to give readers additional information about their work.

© 2025 Radwan RM et al. *JAMA Network Open*.

eTable. Exposures of Interest and Comparison

**Category**	**Medications**
GLP-1RAs	Albiglutide*, Dulaglutide, Exenatide, Liraglutide, Lixisenatide, Semaglutide, Tirzepatide
SGLT2i	Canagliflozin, Dapagliflozin, Empagliflozin, Ertugliflozin, Sotagliflozin, Bexagliflozin
DPP4i	Sitagliptin, Saxagliptin, Alogliptin, Linagliptin

*Withdraw from the market in 2018. GLP-1RAs, glucagon-like peptide-1 receptor agonists; SGLT2i, sodium-glucose cotransporter 2 inhibitors; DPP4i, dipeptidyl peptidase 4 inhibitors.

© 2025 Radwan RM et al. *JAMA Network Open*.
