# Supplementary material for: GLP-1 RA Use and Survival Among Older Adults With Cancer and Type 2 Diabetes
Source: JAMA Netw Open. 2025 Jul 18;8(7):e2521887. doi: 10.1001/jamanetworkopen.2025.21887 (PMC12274974; doi:10.1001/jamanetworkopen.2025.21887)
Supplement: Supplement 2. — Data Sharing Statement [file jamanetwopen-e2521887-s002.pdf]

Data Sharing Statement

Radwan. GLP-1 RA Use and Survival Among Older Adults with Cancer and Type 2 Diabetes. *JAMA Netw Open*. Published July 18, 2025. doi:10.1001/jamanetworkopen.2025.21887

Data
Data available: No

Additional Information
Explanation for why data not available: The data analyzed in this study are subject to the following licenses/restrictions: The Medicare claims data contain patient-level health information and are considered identifiable files. Therefore, access to these data requires a data use agreement. Requests to access these datasets should be directed to resdac@umn.edu.
